# *Aggregatibacter actinomycetemcomitans* NadN contributes to neutrophil extracellular trap degradation

**DOI:** 10.1080/20002297.2026.2694756

**Published:** 2026-07-06

**Authors:** Yaowapa Puanglai, Anongnard Kasorn, Oranart Matangkasombut, Dujduan Waraho-Zhmayev, Soraya Chaturongakul, Fabien Loison

**Affiliations:** a Department of Microbiology, Faculty of Science, Mahidol University, Bangkok, Thailand; b Department of Basic Medical Science, Faculty of Medicine Vajira Hospital, Navamindradhiraj University, Bangkok, Thailand; c Department of Microbiology and Center of Excellence on Oral Microbiology and Immunology, Faculty of Dentistry, Chulalongkorn University, Bangkok, Thailand; d Research Laboratory of Biotechnology, Chulabhorn Research Institute, Bangkok, Thailand; e Biological Engineering Program, Faculty of Engineering, King Mongkut's University of Technology Thonburi, Bangkok, Thailand; f Center for Advanced Therapeutics, Institute of Molecular Biosciences, Mahidol University, Nakhon Pathom, Thailand

**Keywords:** Periodontitis, host-pathogen interaction, virulence factor, nuclease, immune evasion

## Abstract

**Background:**

Neutrophils Extracellular Traps (NET) immobilize and kill extracellular pathogens. *Aggregatibacter actinomycetemcomitans (Aa)*, associated with aggressive forms of periodontitis, has been shown to induce NET release. However, it remains unknown whether *Aa* evades extracellular traps.

**Materials and methods:**

NETs were incubated with wild-type *Aa* (*Aa*-WT, strain Y4) or *Aa*-ΔCdtB, lacking a toxin inducing DNA breaks. NET degradation was measured by imaging and fluorometry. *Aa* survival and growth was measured by biofilm formation. *Aa*-WT membrane was fractionated on a sucrose gradient, and fractions positive for NET degradation were subjected to LC-MS/MS. The top protein candidate was expressed as a recombinant protein in *Lactococcus lactis* to confirm its NET-degrading activity.

**Results:**

*Aa*-WT and *Aa*-ΔCdtB strains were captured by NETs but degraded the extracellular traps after three hours. *Aa* survival in the presence of NETs was independent of CdtB expression. NET degrading activity was not release but associated with cells. NAD nucleotidase (NadN) was identified via mass spectrometry from membrane fraction. *In vitro*, recombinant NadN degraded NETs, and heterologous expression of NadN in *L. lactis* was sufficient to confer a NET-degrading phenotype.

**Conclusions:**

*Aa* degrades NETs to evade host immunity. NadN is a primary mediator of the NET-degrading activity of *Aggregatibacter actinomycetemcomitans.*

## Article highlights

- *A. actinomycetemcomitans* Y4 strain can evade the innate immune response via the degradation of NETs.
- The toxin CdtB is not required for survival of *Aa* in the presence of NETs, nor for NET degradation.
- The membrane-associated NAD nucleotidase (NadN) acts as a primary mediator of NET degradation.

## Introduction

Periodontitis is the result of inflammatory responses to microbial dysbiosis leading to periodontal tissue destruction affecting over 1 billion people worldwide [1]. The etiology of periodontitis is multifactorial, including bacterial, environmental and host factors [2,3]. *Aggregatibacter actinomycetemcomitans* (*Aa*) is a gram-negative member of the HACEK group, associated with aggressive forms of periodontitis, and infective endocarditis, systemic infections and rheumatoid arthritis [4–8]. Periodontal lesions may serve as a portal of entry for oral pathogens to enter the bloodstream [9]. Half of patients with *Aa*-associated endocarditis have a history of dental carries, periodontitis or dental procedure [10]. However, the mechanisms underpinning the transition from localized periodontal lesions to systemic infection are still ill defined.

Neutrophils are the most abundant leukocytes in the blood circulation and are central in the periodontal immune response. They are continuously recruited to the gingival sulcus in response to damages of the junctional epithelial cells [11,12]. There, they eliminate microbes by phagocytosis, degranulation, oxidative burst and the formation of Neutrophil Extracellular Traps, or NETs [13]. NETs, composed of extracellular chromatin decorated with antimicrobial proteins, immobilize pathogens [14,15]. Many bacteria, including periodontal species, express nucleases that degrade NETs and facilitate their immune evasion [16–18]. *Aa* produces multiple virulence factors used to subvert host defense, including the cytolethal distending toxin (CDT), whose subunit, CdtB participates in bacterial dissemination [19,20]. CdtB DNase activity has also been shown to induce DNA damages, cell cycle arrest and cell death [21–23]. While *Aa* has been shown to induce NETs via leukotoxin (Ltx) secretion [9,24], it is unknown whether *Aa* is captured and killed by NETs, or has the ability to evade NETs. To address this knowledge gap, this study aims to (i) measure the survival of *Aa* in the presence of NETs, (ii) test whether the toxin CdtB is an important factor for *Aa* survival in the presence of NETs and (iii) identify other factors participating in NET degradation.

## Materials and Methods

### Bacterial strains

*Aggregatibacter actinomycetemcomitans* (*A. actinomycetemcomitans*) strains, Y4 serotype b (*Aa*-WT) and CdtB mutant (*Aa*-ΔCdtB) were kindly provided by Prof. Motoyuki Sugai, Hiroshima University, Japan. *Aa*-WT and *Aa*-ΔCdtB strains were grown in Brain heart infusion (BHI; BD #211059) broth, or agar supplemented with 5% (v/v) fetal bovine serum (FBS) at 37 °C, 10% CO_2_. BL21(DE3) *E. coli* was grown in Luria-Bertani (LB) broth (BD #244620). *Lactococcus lactis* NZ9000 (*L. lactis*, MoBiTec GmbH, Germany) was cultured in M17 broth (Himedia #M1029) or agar supplemented with 0.5% (w/v) glucose at 30 °C.

### PKH26 labeling of *A. actinomycetemcomitans*

Overnight cultures were washed once with sterile with phosphate buffered saline (PBS), and the bacterial pellets were labeled using the membrane dye PKH26 following the manufacturer's protocol (4 μM, Sigma #PKH26GL). Labeled *Aa* pellets were washed twice with PBS, and the staining efficiency (% of stained bacteria) was measured by flow cytometry (CytoFLEX, Beckman Coulter, Inc). Bacterial viability was determined by culturing serial dilutions of the stained bacteria on agar plates and determination of the colony-forming units (CFU).

### Neutrophil isolation from peripheral blood

The project was approved by the Center of Ethical Reinforcement for Human Research, Mahidol University (COA No. MU-CIRB 2022/283.2010). Blood samples were collected from healthy volunteers with informed consent. Neutrophils were isolated using a previously published protocol [25]. After isolation, neutrophils were resuspended in Roswell Park Memorial Institute (RPMI) medium without antibiotics. Neutrophils purity and activation state were assessed using flow cytometry.

### NET induction and degradation

Sterile, round, 12 mm cover glasses were added to the bottom of the wells in a 24-well plate. Purified primary neutrophils were seeded (2 × 10^5^ cells per well) in 500 µl of RPMI (+2% autologous serum), and pre-incubated at 37 °C, 5% CO_2_ for 30 minutes to enable neutrophil adhesion. NET formation was stimulated overnight by the addition of phorbol 12-myristate 13-acetate (PMA, 100 nM, Sigma #P8139). Medium containing PMA was removed and replaced with either RPMI containing PKH26 labeled-*Aa*-WT or -ΔCdtB, or purified recombinant proteins. Fresh RPMI was added to NETs as a negative control. DNase I (4 units/ml, Roche #04716728001) was added to RPMI as a positive control of NET degradation. Following incubation, the coverslips were carefully washed with PBS, and cells were fixed (4% paraformaldehyde) and stained for immunofluorescence.

### Biofilm mass quantification

Biofilm quantification was performed using a protocol adapted from O'Toole et al. [26]. At various time points, supernatants of bacteria-NETs co-incubation were discarded, and the wells were washed twice with sterile water. Biofilms were stained with 0.1% crystal violet for 15 minutes, washed 3‒4 times with tap water, and dried overnight. The stained biofilm mass was solubilized (125 µl of 30% acetic acid, 15 minutes) and transferred to a 96-well plate for quantification by measuring the absorbance at 595 nm (EZ Read 400 Microplate Reader, Biochrom, USA), using 30% acetic acid in water as the blank.

### Immunofluorescence staining of NETs and *A. actinomycetemcomitans*

Coverslips were washed with PBS, fixed with 4% paraformaldehyde and blocked for 30 minutes (3% bovine serum albumin and 5% human serum in PBS). Staining with the primary mouse anti-myeloperoxidase antibody was done overnight at 4 °C (1:500, Santa Cruz Biotechnology #sc-52707), followed by washes and a 1-hour incubation with secondary anti-mouse IgG-F(ab)2 conjugated with Alexa 488 (1:2000, Cell Signaling Technology #4408). DNA was detected with Hoechst 33342 (15 minutes, 5 µM of Invitrogen, #H21492). Specimens were mounted with Antifade Mounting (Invitrogen #P10144), and pictures were taken using an epifluorescence microscope (Olympus BX53 Fluorescence microscope).

### NET isolation

NETs were induced as described above and were isolated as previously described [27]. Briefly, NETs were released from the well by mild digestion with the restriction enzyme Alu I at 4 U/ml for 20 minutes at 37 °C (New England Biolabs # R0137S). Supernatants were collected and centrifuged (300 g, 4 °C, 5 minutes) to remove cellular debris. The presence of NETs in the supernatants was confirmed by gel electrophoresis (0.8% agarose).

### NET quantification

In imaging experiments, the area covered by NETs was quantified using a method previously described [28,29]. Briefly, using ImageJ 1.53, the area of at least 50 regions of interest (ROI) was quantified. NET-negative cells were morphologically detected as small, round, or lobulated nuclei, while NETs were web-like structure. Images were converted to 8-bit grayscale, the image threshold set and the area of particles (μm^2^) analyzed. Fragments or cell debris with area smaller than 300 μm^2^ were excluded. The ‘analyze particle’ function was used to export information on each ROI into a .csv file for further analysis. NET area was normalized with NET control condition and quantified using the following formula:

$$\%\;\text{NET area}=\left(\frac{100}{\text{area of NET ctrl}}\right)\;\times \;\text{area of NETs with bacteria or DNase}$$

For the quantification of NETs using microplate assay, isolated NETs (0.2 µg of DNA) were incubated in DNase buffer (10 mM Tris-HCl, pH 7.5, 2.5 mM MgCl_2_, 0.5 mM CaCl_2_) with *Aa* cell-free conditioned medium, membrane extract or recombinant proteins, in a 1:1 ratio with the 1:200 dilutions of Quant-iT™ PicoGreen™ dsDNA reagent (Invitrogen #P7581) using black 96-well plate (Corning #33603). The reaction that was incubated in the presence of Ethylenediaminetetra acetic acid (EDTA) (F_EDTA_) was used as the negative control and for normalization. Fluorescence intensity was measured every hour for 6 hours (37 °C, excitation wavelength of 485 nm, emission wavelength of 535 nm (Spark multimode microplate reader, Tecan Trading AG, Switzerland)). The blank value (DNase reaction buffer with PicoGreen) was subtracted from the sample's fluorescence value. NET degradation was determined by the following formula:

$$\%\;\text{NET degradation}=100\;\times \;(\frac{{\text{F}}_{\text{EDTA}}\;-\;\text{F}}{{\text{F}}_{\text{EDTA}}})$$

where F_EDTA_ and F were the average fluorescence signals of the experiment with and without EDTA, respectively. All experiments were repeated at least three times, performed in triplicates, using three independent human neutrophil samples.

### *A. a* *ctinomycetemcomitans* membranes extraction

Membrane extractions from *Aa* was performed as previously published [30]. Briefly, log phase culture *Aa* pellets were incubated with lysozyme and subjected to osmotic lysis. Then, cell suspensions were centrifuged (200,000 g, 2 hours, 4 °C) using a 50.2 Ti rotor (Beckman Instruments Inc.). Membrane pellets were resuspended in a modified suspension buffer (25% w/w sucrose, 5 mM Tris, 30 mM MgCl_2_, 1 tablet of EDTA-free protease inhibitor cocktail (Roche #5892791001). Next, membrane suspensions were layered on top of a discontinuous gradient solution (55%, 50%, 45%, 40%, 35% and 30%, 600 µl each), and centrifuged at 256,000g (18 hours, 4 °C) in a SW 55 Ti rotor (Beckman Instruments Inc.). Proteins were concentrated from fractions (500 µl) collected sequentially from the top layer using Amicon Ultra-0.5 mL Centrifugal Filters 10KDa cut off (Merck Millipore # UFC5010). The protein content was measured by Bicinchoninic Acid (BCA) assay (Pierce™ BCA Protein Assay Kit, Thermofisher Scientific).

### Sodium dodecyl sulfate polyacrylamide gel electrophoresis (SDS-PAGE) and silver staining

SDS gel electrophoresis of proteins was performed using standard procedures and detected by silver staining. Briefly, gels were fixed overnight in the fixing solution (40% v/v ethanol, 10% v/v acetic acid, 50% water), and next incubated with the pre-treatment solution for 30 minutes with agitation (0.2% w/v sodium thiosulfate, 6.8% w/v sodium acetate, 30% v/v ethanol, 0.125% v/v glutaraldehyde). After 3 washes with water, gels were submerged in the silver solution for 30 minutes (0.25% w/v silver nitrate (AgNO_3_), 0.015% v/v formaldehyde), before being washed twice more. Silver staining was allowed to develop for 3‒5 minutes (2.5% w/v sodium carbonate (Na_2_CO_3_), 0.0074% v/v formaldehyde) before the addition of the stop solution (1.5% w/v EDTA). All steps were performed at room temperature and under agitation. Images of the silver-stained gels were captured using ChemiDoc XRS +  (BIO-RAD).

### Western blot analysis

The proteins or fractions of interest were separated in 10% SDS–PAGE gels before being transferred to a 0.45 µm PVDF membrane (Merck Millipore, Germany). The polyvinylidene fluoride (PVDF) membranes were blocked for 30 minutes at room temperature (5% skimmed milk in TBS-T; Tris-buffered saline, 0.1% Tween 20). The primary antibody (rabbit anti-CdtB, kindly gifted by Prof. Motoyuki Sugai, or rabbit polyclonal anti-6xHistidine antibody (Cell Signaling Technology #2365)) was incubated overnight at 4 °C. After 3 washes in TBS-T, membranes were incubated with the HRP-conjugated secondary antibody for 1 hour at room temperature (anti-rabbit IgG, HRP-linked Antibody, Cell Signaling Technology #7074). Chemiluminescence was measured after incubation of the PVDF membranes with ECL Western Blotting Detection Reagent (BIO-RAD #1705061) for 5 minutes, and images were acquired using ChemiDoc XRS + (BIO-RAD).

### PCR analysis

Genes of interest were identified using Blast (NCBI) and the genomic information for *Aa* Y4 Scfld51 (NZ_KB290906.1) in the NCBI database. Regular PCR was performed as follow: initial denaturation at 94 °C for 5 minutes, followed by 35 cycles of amplification (denaturation at 94 °C for 30 seconds, annealing at 59 °C for 30 seconds, extension at 72 °C for 1 minute) and a final extension at 72 °C for 10 minutes. For cloning, Q5® High-Fidelity (NEB #M0491) was used, following these steps: initial denaturation at 98 °C for 30 seconds, 35 cycles of amplification (denaturation at 98 °C, 10 seconds; annealing at 72 °C for 30 seconds; extension at 72 °C for 1 minute) and an additional final extension step at 72 °C for 2 minutes. PCR products were run on 1.8% agarose gel electrophoresis.

### Identification of putative nuclease by LC-MS/MS

*Aa* membrane fraction number 8 was precipitated using a 1:5 (v/v) ratio of acetone and 5 mM Dithiothreitol (DTT) at −20 °C for 3 hours. Protein pellets were collected by centrifugation (10,000 g, 10 minutes, 4 °C) and air dried. The digestion buffer (0.2% RapiGest SF Surfactant (Waters), 10 mM NaCl, and 10 mM ammonium bicarbonate) was added to solubilize the protein pellet. Protein pellets were sonicated at a frequency of 20 kHz and 80% amplitude for 2 seconds, and the protein concentration was adjusted to 1 µg/µl. Subsequently, samples were incubated at 90 °C for 15 minutes in DTT solution (20 mM DTT in 10 mM ammonium bicarbonate; final concentration 5 mM DTT). Next, samples were incubated in the dark (RT, 25 minutes) before a cleaned up using Zeba Spin Desalting Columns (ThermoFisher Scientific). Proteins were digested for 4 more hours (0.5% RapiGest SF, 100 ng/μl Trypsin), and the reaction was stopped with 1% formic acid (1:40 (v/v) ratio). Tryptic peptides were then lyophilized and subjected to LC-MS/MS analysis. LC-MS/MS spectrums were collected in the positive mode on an Orbitrap HF mass spectrometer combined with a nano-LC system equipped with an EasySpray C18 column (ThermoFisher Scientific). Raw LC–MS/MS files were subjected for analysis using the Proteome Discoverer with SEQUEST™ HT algorithm (ThermoFisher Scientific), referencing the reviewed UniProt *Aa* database.

### Expression and purification of the recombinant NadN protein (rNadN)

The plasmid pET28a-NadN-6xHis (7.153 kb) was custom-synthesized by GenScript as follows. The *nadN* gene (codon-optimized for *E. coli*) was cloned into the pET28a backbone between the EcoRI and XhoI sites, with a C-terminal His-tag. NadN protein expression was induced in *E. coli* BL21(DE3) with 2 mM of isopropyl-b-D-thiogalactopyranoside (Sigma #5810) at 37 °C for 8 hours, under agitation. Cells were harvested by centrifugation (7,000 g, 10 minutes, 4 °C) and washed with 10 mM Tris pH 7.5. The pellet was resuspended in lysis buffer (50 mM NaH_2_PO_4_, 300 mM NaCl, 10 mM imidazole, pH 8) and sonicated thrice on ice (amp. 40%, on 10 sec/off 10 sec). Cell debris were removed from the crude lysate via centrifugation (15,000 g, 30 minutes, 4 °C). The expression of recombinant NadN was confirmed by SDS-PAGE followed by Coomassie staining and western blot using anti-6xHistidine antibodies. The recombinant NadN-6xHis was purified from the soluble crude lysate under native conditions using Ni-NTA agarose (MACHEREY-NAGEL #745400.25).

### NadN expression in Lactococcus lactis NZ9000

*NadN*-6xHis gene was subcloned in pNZ8120 vector (MoBiTec GmBH) containing a nisin-inducible promoter (PnisA) and chloramphenicol resistance gene (CmR). Briefly, the gene was amplified via PCR, inserting the restriction sites NaeI and XbaI. After transformation via electroporation (2000 V, 25 μF, 200 *Ω*), transformed *L. lactis* were incubated in M17 broth supplemented with 0.5% glucose, 20 mM MgCl_2_ and 2 mM CaCl_2_ for 1.5 hours at 30 °C, and then plated on M17 agar + 0.5% glucose + 10 μg/ml Chloramphenicol (Sigma #C0378). After 48 hours at 30 °C without agitation, CmR resistant *L. lactis* clones were selected and isolated plasmids were sequenced (U2Bio, Korea). Overnight cultures of *L. lactis* were diluted in fresh media and grown until OD600 reached 0.4 and, NadN expression was induced with 10 ng/ml of Nisin (Sigma #N5764).

### Statistical analysis

Statistical analyzes were performed using GraphPad Prism version 6.01 for Windows. The T-test with the Holm-Sidak correction was used to compare the mean between two groups of treatment (e.g. without DNase vs. with DNase). Differences between more than two groups of samples were determined by one-way ANOVA, followed by Turkey's multiple comparisons tests.

## Results

### A. actinomycetemcomitans co-localized and degraded NETs in vitro

**Figure 1.**
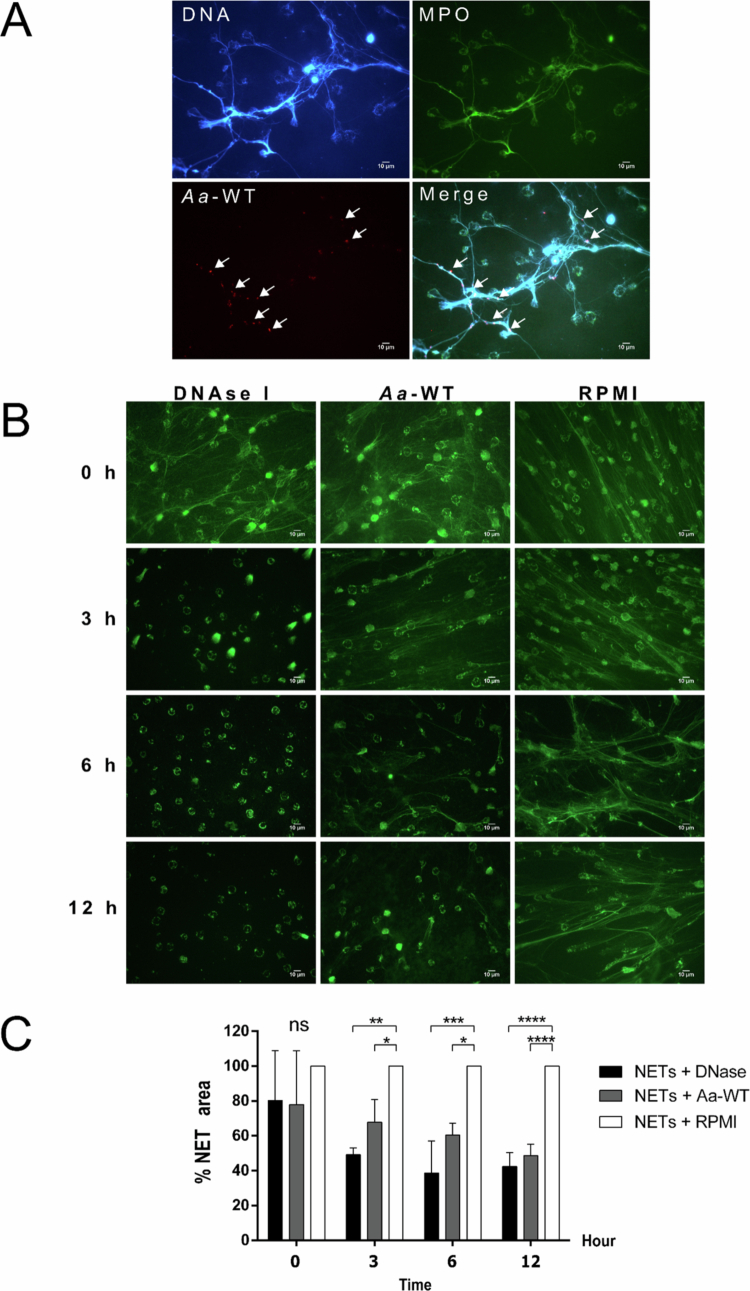
*A. actinomycetemcomitans* capture by neutrophil extracellular traps was followed by NET degradation. (A) Representative immunofluorescence microscopy pictures demonstrating the co-localization of *Aa* within the lattice structure of NETs. (Scale bar = 10 μm; Magnification: 400×; MPO: green, DNA: blue, bacteria: red and white arrows). (B) Immunofluorescence images of NETs following co-incubation either with DNase I (4 U/ml, positive control), *Aa*-WT (MOI = 4), or with RPMI alone (negative control). Representative pictures taken at 0, 3, 6 and 12 hours. (C) Quantitative analysis of NET surface area was performed using ImageJ software. Data were presented as mean + /− SD (*n* = 3). Statistical significance was determined by one-way ANOVA followed by Tukey's multiple comparisons test; **** *p* < 0.0001; ns, non-significant.

We first tested whether *Aa* was trapped in NETs. Live labeled *Aa* (Figure S1) was initially associated with human neutrophils derived NETs, as evidenced by their co-localization with DNA and myeloperoxidase (MPO) (Figure 1A). Next, NETs were cultured for up to 12 hours with DNase I or with the *Aa* Y4 serotype b wild-type strain (*Aa-*WT). As shown in Figure 1B, NETs remained structurally intact in RPMI alone (negative control, right column), while the extracellular fibers were degraded in three hours when DNase I was added to the medium (positive control, left column). Interestingly, incubation of *Aa* WT with NETs resulted in the progressive loss of fibrous chromatin structures after three hours (Figure 1B, middle column). Quantitative analysis of the area covered by the NETs confirmed that *Aa-*WT significantly reduced the NETs network to levels comparable to the DNase I positive control by 12 hours (~40% reduction, Figure 1C). These data comforted our hypothesis that *Aa* is capable of NET degradation.

### *A. actinomycetemcomitans* degradation of NETs was independent of the cytolethal distending toxin

**Figure 2.**
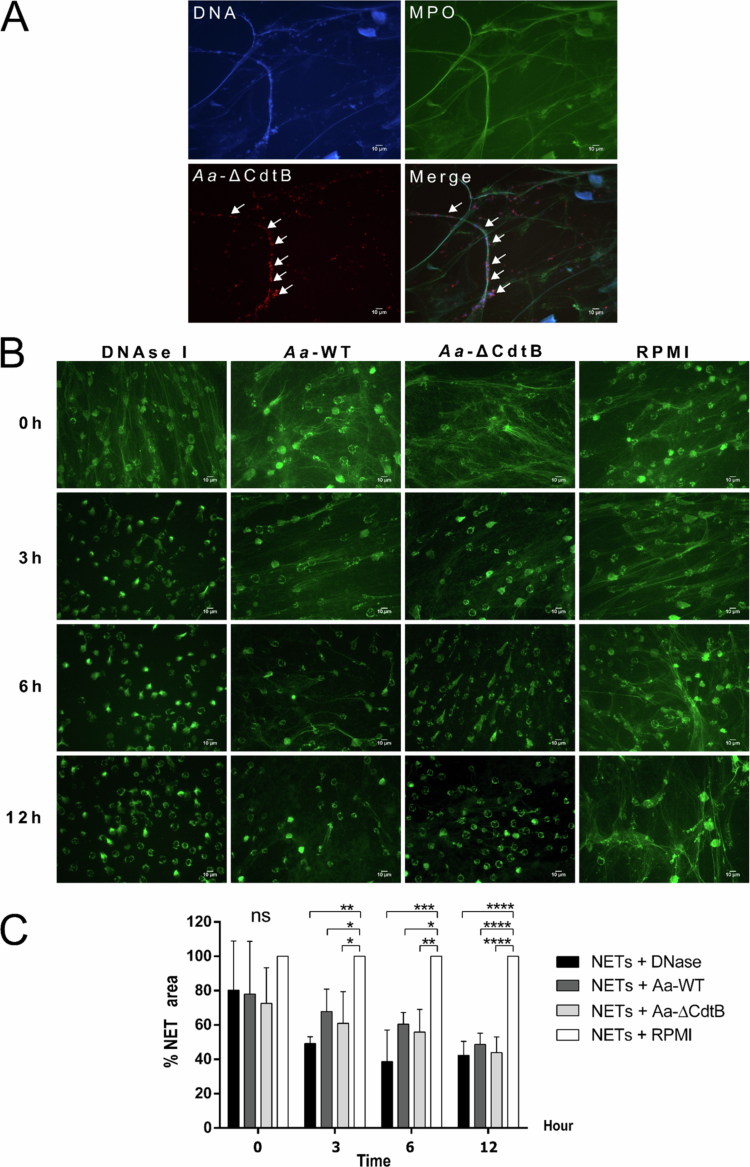
NET degradation by *A. actinomycetemcomitans* occurred independently of CdtB expression. (A) Immunofluorescence microscopy of NETs following co-incubation with the *Aa*-ΔCdtB mutant strain, illustrating bacterial co-localization within the NET structure. (Scale bar = 10 μm; Magnification: 400×; MPO: green, DNA: blue, bacteria: red, white arrows). (B) Immunofluorescence images of NET degradation following co-incubation either with DNase I (4 U/ml, positive control), *Aa*-WT, *Aa*-ΔCdtB or with RPMI alone (negative control). Representative pictures taken at 0, 3, 6 and 12 hours. (C) Quantification of NET surface area performed using ImageJ software. Data were expressed as mean + /− SD (*n* = 3). Statistical significance was evaluated by one-way ANOVA followed by Tukey's post hoc multiple comparisons test; ****p < 0.0001; ns, non-significant.

Next, we investigated whether *Aa*'s CdtB could serve as the effector molecule responsible for NET degradation, utilizing a CdtB mutant strain of *A. actinomycetemcomitans* (*Aa*-ΔCdtB, Figure 2 and S2). Co-incubation experiments revealed that, similarly to *Aa-*WT, *Aa*-ΔCdtB was initially captured in NETs generated *in vitro* (Figure 2A). Interestingly, *Aa*-ΔCdtB retained the capacity to degrade NETs in a time-dependent manner, with no statistically significant difference in degradation efficiency compared to the WT strain (Figure 2B–C). Altogether, these results indicated that *Aa* employed a CdtB-independent strategy for NET degradation, implying the expression of alternative nucleases.

### *A. actinomycetemcomitans* WT and ΔCdtB strains were able to evade NETs capture

**Figure 3.**
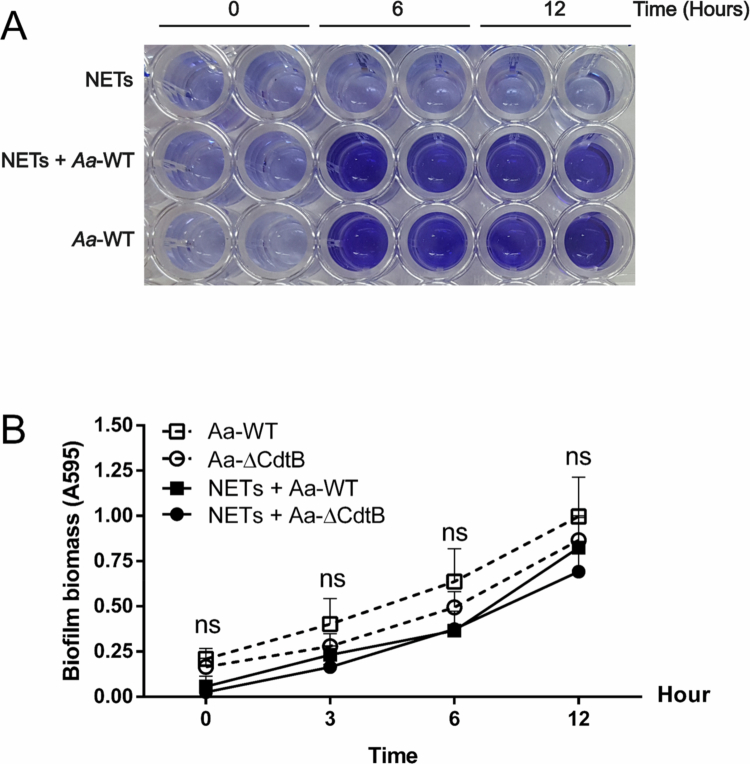
*A. actinomycetemcomitans* biofilm formation was not impaired by the presence of NETs. (A) Representative picture of the crystal violet staining of *A. actinomycetemcomitans* biofilm (WT and ΔCdtB, MOI = 4) at time 0, and after exposure to NETs for 6 and 12 hours. The stained biofilm mass was washed, dried, solubilized, and transferred to a 96-well plate. (B) The absorbance at 595 nm was measured at time 0, and after 3, 6 or 12 hours of co-incubation with NETs. Data were normalized to the NETs-only control group. Mean + /− SD (*n* = 3). Statistical analysis was performed using multiple t-tests with the Holm‒Sidak correction for multiple comparisons; ns, non-significant (*p* > 0.05).

To confirm these findings, we evaluated the impact of NETs on bacterial viability by quantifying biofilm formation of *Aa* -WT and *Aa*-ΔCdtB *in vitro* in the presence and the absence of NETs. Of note, both strains exhibited comparable growth kinetics in the absence of NETs (Figure S2C). Although a slight reduction in biofilm biomass produced by *Aa*-WT was observed in the presence of NETs compared to the control, this trend did not reach statistical significance (Figure 3A and B). Furthermore, no significant difference in biofilm production was observed between the *Aa*-WT and *Aa*-ΔCdtB strains (Figure 3B), suggesting that *Aa* survival and growth were not altered by the presence of NETs, and were independent of CdtB expression.

### *A. actinomycetemcomitans* nuclease activity was not detected in conditioned media

As several bacterial species secrete extracellular factors to neutralize NETs [18], we investigated whether *Aa* secreted nuclease activity by establishing a cell-free fluorescence-based assay. To establish the assay, the fluorescent dye Picogreen was used to quantify the degradation of lambda DNA and purified NETs by DNase I in a time- and dose-dependent manner (Figure S3). *Aa* cell-free conditioned media was harvested from culture in complete RPMI (without phenol red) at 0, 12 and 24 hours, corresponding to the lag, log and the stationary phase, respectively (Figure S2C) and concentrated up to 8-fold (Table S1). However, no significant degradation of NETs was noticeable following incubation with the concentrated conditioned media (Figure S4). These results suggested that the observed nuclease activity was likely mediated by protein(s) associated with the bacterial cell membrane.

#### The NET-degrading activity was detected in the membrane fraction of *A. actinomycetemcomitans*

**Figure 4.**
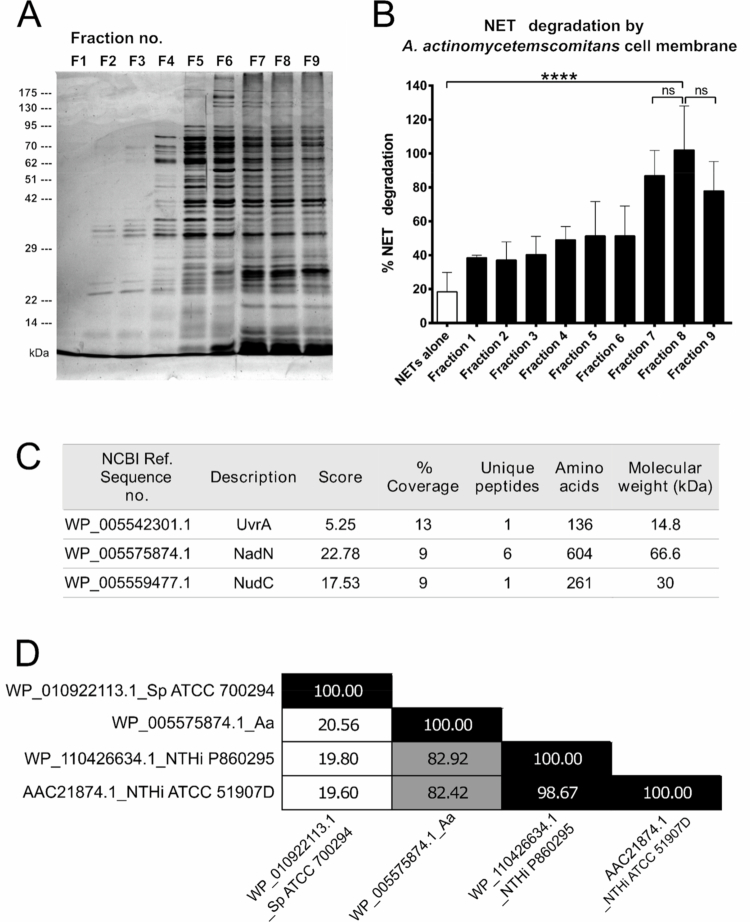
Identification of NadN as a putative nuclease within *A. actinomycetemcomitans* (*Aa*) membrane fractions. (A) Silver-stained 10% SDS-PAGE gel showing the protein profile of *Aa* cell membrane fractions F1 to F9. Each lane was loaded with 20 ng of protein. (B) Measurement of the NET degradation activity in each isolated membrane fraction using 150 ng of protein via the PicoGreen fluorescence-based assays. Mean ± SD, *n* = 4. ****p < 0.0001 (ANOVA - Tukey's multiple comparisons test). (C) Identification of the top three putative nuclease candidates in fraction 8 following liquid chromatography-tandem mass spectrometry (LC-MS/MS). (D) Pairwise sequence identity (%) matrix of NadN homologs by multiple sequence alignment (MSA) using Clustal Omega. *A. actinomycetemcomitans* (Acc. No. WP_005575874.1; current study), *Streptococcus pyogenes* ATCC 700294 (Acc. No. WP_010922113.1), *Haemophilus influenzae* strain P860259 (Acc. No. WP_110426634.1) and *H. influenzae* ATCC 51907D (Acc. No. *AA*C21874.1).

To test our hypothesis, *Aa* membrane suspensions were subjected to sucrose gradient ultracentrifugation [30], yielding nine distinct fractions (F1–F9). SDS-PAGE followed by silver staining revealed protein profiles for each fraction (Figure 4A). Each fraction was concentrated using molecular weight cut-off (MWCO) filtration, as previously described. Subsequent functional screening via PicoGreen assay demonstrated that while the whole-cell lysate exhibited negligible DNase activity at equivalent protein concentrations, the membrane suspension effectively degraded NETs (Figure S5). High-potency NET degradation was detected in fractions 7, 8 and 9, with fraction 8 exhibiting the most robust activity (*p* < 0.0001 compared to control, Figure 4B). To identify the protein(s) involved in the degradation of NETs, fraction no. 8 was subjected to LC-MS/MS. Among the proteins with nuclease-related functions identified, a putative *Aa* NAD (nicotinamide adenine dinucleotide) nucleotidase appeared as the most prominent candidate (WP_005575874.1, Figure 4C). NAD nucleotidase (NadN), also known as 5′ nucleotidase (5Nuc), facilitates the hydrolysis of nucleotides by cleaving phosphate group from the 5′-position of the pentose ring [31]. Sequences alignment showed that *Aa*-NadN and *Hi*-NadN from *Haemophilus influenzae* strain P860259 and *H. influenzae* strain ATCC 51907D share ~82% sequence identity (Figure 4D). However, Aa-NadN shared only ~20% sequence identity with the homologous *Streptococcus pyogenes* ATCC 700294 (Figure 4D).

#### Recombinant NadN was able to degrade NETs

**Figure 5.**
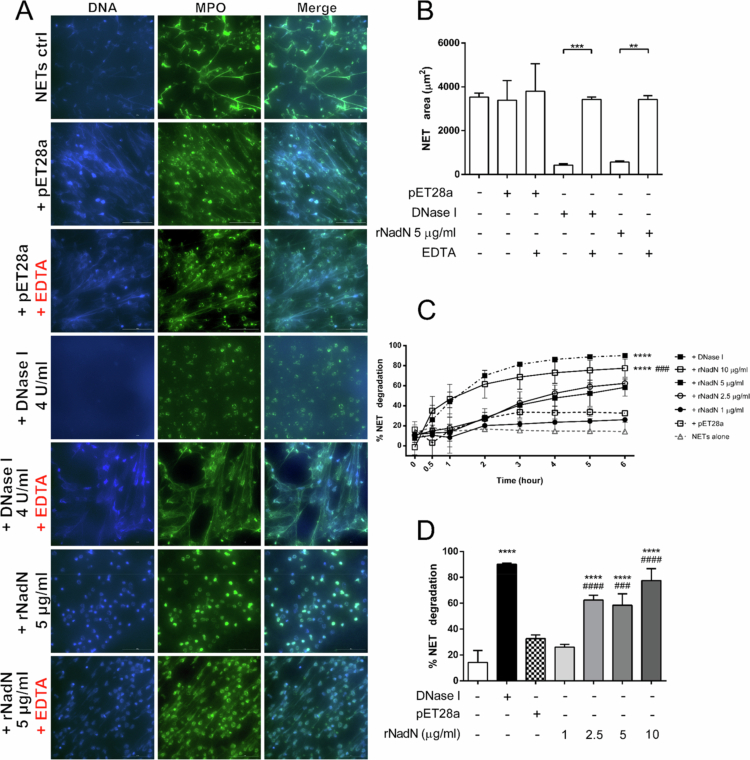
Recombinant NadN (rNadN) mediates the degradation of NETs. (A) Representative immunofluorescence images after co-incubation for 6 hours of NETs (MPO: green; DNA: blue) with rNadN, DNase I (positive control), or pET28a vector (negative control), with or without EDTA. Scale bar = 100 μm; magnification: 400×. (B) Image-based analysis of NET area. Mean + /− SD (*n* = 3). (C) Kinetic of NET degradation using a PicoGreen fluorescence-based microplate assay. Mean + /− SD (*n* = 3). (D) Percentage of NET degradation at the 6-hour time point as determined by PicoGreen assay. Mean + /− SD (*n* = 3). Statistical significance was assessed by one-way ANOVA followed by Tukey's multiple comparisons test. (* vs. NETs alone; # vs. NETs + pET28a control; **** or #### *p* < 0.0001; *** or ### *p* < 0.0005).

To functionally validate the role of NadN, the gene was amplified from *Aa*-WT genomic DNA and cloned into the pET28a expression vector, incorporating a C-terminal 6xHis tag (Figure S7A). Protein expression was confirmed via nickel-nitrilotriacetic acid (Ni-NTA) affinity chromatography and subsequent anti-6xHis immunoblot following Isopropyl *β*-d-1-thiogalactopyranoside (IPTG) induction. Two distinct immunoreactive bands were detected at 67 and 60 kDa (Figure S7C), likely corresponding to the immature and mature isoform of NadN as reported in *H. influenza* [32]. Incubation of human NETs with recombinant NadN (rNadN) resulted in the complete destruction of the extracellular fibrous chromatin structures within 6 hours as compared to the negative control (NETs alone) (Figure 5A–B). NETs incubated with eluates from cells transformed with the empty pET28a vector remained intact, excluding the possibility of contamination during the purification process (pET28a, Figure 5A–B). Furthermore, the DNase activity of purified rNadN was validated using a PicoGreen-based fluorometric assay. EDTA was used as negative control, as divalent cations calcium and magnesium are required for DNase I and NadN nuclease activity [33,34]. In the presence of EDTA, both enzymes were unable to degrade NETs *in vitro* (Figure 5A and B). The digestion of NETs over time followed a dose-dependent response to rNadN concentration (1 to 10 µg/ml) (Figure 5C and D). The maximum NET degradation was observed at a concentration of 10 µg/ml, whereas the negative controls (NETs alone and pET28a) exhibited negligible background degradation over the 6-hour incubation (Figure 5D).

#### Heterologous NadN expression was sufficient to mediate NET degradation

**Figure 6.**
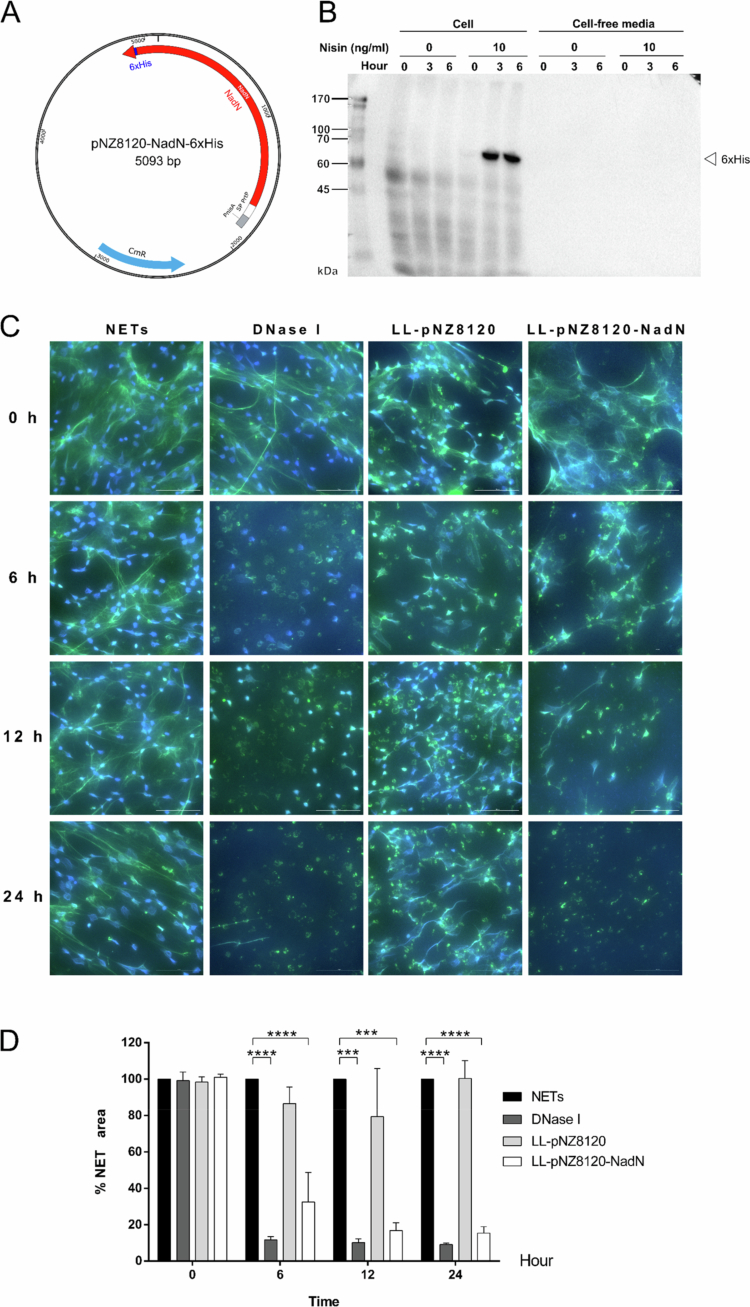
Heterologous expression of *Aa*-NadN in *Lactococcus lactis* (LL) was sufficient to mediate NET degradation. (A) pNZ8120-NadN-6xHis expression vector. (B) Western blot analysis of NadN expression in *L. lactis* whole-cell lysates and cell-free supernatants using anti-6xHis antibody (◁). (C) Representative immunofluorescence images of NETs incubated in RPMI alone (NETs), or co-incubated with DNase I (positive control), LL-pNZ8120 (*L. lactis* + empty vector, negative control), or LL-pNZ8120-NadN (*L. lactis* + pNZ8120-NadN-6xHis). Co-incubation with bacteria was done at MOI = 200. Scale bar = 100 μm; Magnification: 400x; MPO: green, DNA: blue. (D) Quantification of (C), using ImageJ. Data represented as mean + /− SD (*n* = 3). Statistical significance was determined by one-way ANOVA followed by Tukey's multiple comparisons test. (****p < 0.0001; ***p < 0.0005 vs. NETs control at the corresponding time point).

To explore whether NadN expression was sufficient to confer NET evasion capability, we use the heterologous expression system in *Lactococcus lactis* [35]. The *nadN* gene was cloned into the pNZ8120 vector under the control of the nisin-inducible PnisA promoter, featuring an *N*-terminal PrtP signal peptide for membrane targeting and a C-terminal 6xHis-tag (pNZ8120-NadN, Figure 6A). Following nisin induction, immunoblotting confirmed the expression of recombinant NadN. The PrtP signal peptide successfully mediated the translocation of the protein to the lactococcal membrane [36], as evidenced by the absence of 6xHis-tagged protein in the concentrated culture supernatant (Figure 6B). Upon co-incubation with human PMA-induced NETs, *L. lactis* expressing NadN effectively abolished the fibrous chromatin structures of NETs within 24 hours, as compared to the negative control (Figure 6C and D). Conversely, NETs remained structurally intact when incubated with *L. lactis* harboring the empty pNZ8120 vector, providing definitive evidence that NadN expression was sufficient to facilitate bacterial degradation of NETs, and potentially enabling the escape from the NET-mediated entrapment.

## Discussion

Neutrophil extracellular traps (NETs) are abundant in gingival sulci and represent an important antimicrobial defense [12,37,38]. These DNA-based structures immobilize and kill microorganisms through associated antimicrobial proteins [14,39]. However, many periodontal pathogens have evolved mechanisms to resist NET-mediated killing, including the secretion of extracellular nucleases that degrade the NETs scaffold [18,40,41]. In this study, we identify NadN from *Aa* as an enzyme participating in the degradation of NETs *in vitro* and demonstrate that NET degradation occurs independently of the cytolethal distending toxin DNase subunit CdtB. In parallel to the ability to destroy NETs, bacteria also release molecules that trigger the release of extracellular traps [42]. In the case of *Aa*, it has been postulated that LtxA could induce NETosis, which may contribute to its role in the pathogenesis of rheumatoid arthritis [8,43]. Whether or not NadN is part of *Aa*'s ‘countermeasure’ toolset would be an interesting avenue to explore.

Because of the well-characterized DNase activity of CdtB, we initially hypothesized that it might mediate NET degradation. However, NET degradation occurred in the presence of both a wild-type *Aa* and a CdtB mutant strain, indicating that CdtB was not solely responsible for this activity. Moreover, NET exposure did not impair biofilm biomass for either strain, suggesting that *Aa* resisted NET-mediated killing independently of CdtB. Interestingly, in other members of the *Pasteurellaceae*, including *Glaesserella parasuis*, degraded NETs promoted bacterial growth [44]. NET degradation was also used to generate metabolites that supported bacterial proliferation. Nucleases from *Streptococcus suis* degraded NETs into NAD, supporting the growth of *Actinobacillus pleuropneumoniae* [45]. These observations support a model in which *Aa* uses nucleases to degrade NETs. However, whether *Aa* utilizes the by-products from NET degradation remains to be tested.

Proteomic analysis of *Aa* membrane fractions identified NadN as a candidate nuclease associated with NET degradation. NadN is a 5′-nucleotidase involved in nucleotide hydrolysis and NAD metabolism. Originally characterized in *Haemophilus influenza* (*Hi*) [46], NadN participates in NAD and NADP utilization pathways together with the e(P4) protein. *Hi*-NadN exhibits both NAD pyrophosphatase and NMN 5′-nucleotidase activity, converting NAD to nicotinamide mononucleotide and subsequently to nicotinamide riboside [47]. *Aa-*NadN shares approximately 82% sequence identity with the *Hi* homolog, suggesting functional conservation. In *H. influenzae*, NadN has been detected on the bacterial surface and may adopt a periplasmic or membrane-associated orientation [48]. Sequence analysis of *Aa*-NadN revealed a putative signal peptide suggesting a similar localization (Figure S6), in accordance to a previous report [49].

Bacterial virulence factors can be delivered via outer membrane vesicles (OMVs) released from Gram-negative bacteria through processes associated with envelope stress and membrane remodeling [50,51]. In fact, NadN has been detected in OMVs purified from *Aa,* together with LtxA and CDT [52–54]. A NAD nucleotidase was also reported in the secretome of *Aa* strain D7 [49]. These observations suggest that OMVs may contribute to the extracellular delivery of nucleases. However, in the present study, conditioned media subjected to freeze–thaw and sonication did not exhibit detectable DNase activity. This may be due to low OMV yield in our culture conditions, or to the disruption of vesicle integrity during processing rather than true absence of secreted nucleases. Previous reports used secreted proteins from *Aa* biofilm, or OMVs from *Aa* grown on blood agar plates or stationary phase culture, while we used planktonic log-phase culture [49,52,53]. OMVs from pathogens can bind neutrophils and modulate NETs formation [55,56], raising the possibility that *Aa* OMVs both induce and degrade NETs. Indeed, a recent study showed that *Aa* OMVs could be trapped on NETs [53]. NET-associated histones and antimicrobial peptides could destabilize OMVs, releasing luminal enzymes such as NadN. Although speculative, this model warrants further investigation.

We also employed both recombinant *Aa*-NadN and heterologous expression in *L. lactis* to confirm NadN's NET-degrading activity. The purified protein had a molecular weight of 60–70 kDa, consistent with the predicted molecular weight and homologous proteins in related species. *In vitro* assays demonstrated that recombinant NadN, and *L. lactis* expressing NadN, degraded NETs in a time-dependent manner, as measured by PicoGreen fluorescence. These findings are consistent with previous reports showing that 5′-nucleotidases and related nucleases from diverse bacteria degrade extracellular traps. For example, 5Nuc from *Streptococcus equi* subsp. zooepidemicus degrades NET DNA and generates deoxyadenosine, which suppresses macrophage phagocytosis [57]. Extracellular nucleases from *Streptococcus pyogenes* and other pathogens similarly contribute to immune evasion by dismantling NETs [33,58]. Together, these results support the conclusion that NadN represents a previously unrecognized *Aa* nuclease capable of degrading NETs.

Nevertheless, we recognize that the present study still carries certain limitations. NET degradation was examined primarily using *in vitro* assays and recombinant proteins, and the *in vivo* contribution of NadN to *Aa* virulence remains to be determined. Periodontal infections occur within multispecies biofilms, and interactions among oral microorganisms may influence NET formation and degradation. Future studies should therefore evaluate NadN function in multispecies biofilm models and in animal models of periodontitis. Furthermore, the regulation of NadN expression, as well as the roles it might play in cooperation with CdtB and LtxA in the NET lysis, remain to be seen.

## Conclusions

In summary, we identified NadN as a NET-degrading nuclease produced by *Aggregatibacter actinomycetemcomitans*. NET degradation occurred independently of CdtB, indicating that multiple nucleases may contribute to *Aa* immune evasion. By degrading NETs, NadN may facilitate bacterial survival and potentially generate metabolites that support growth. These findings expand current understanding of *Aa* virulence mechanisms and highlight bacterial nucleases as potential therapeutic targets in periodontal disease.

## Supplementary Material

YP_Aa manuscript_Supplement_TF.pdf YP_Aa manuscript_Supplement_TF.pdf

## Data Availability

Data are available from the authors upon reasonable request to the corresponding author.

## References

[1] Bernabe E , Marcenes W , Abdulkader R , et al. Trends in the global, regional, and national burden of oral conditions from 1990 to 2021: a systematic analysis for the Global Burden of Disease Study 2021. Lancet. 2025;405:897–910. doi: 10.1016/S0140-6736(24)02811-3 40024264

[2] Lamont RJ , Koo H , Hajishengallis G . The oral microbiota: dynamic communities and host interactions. Nat Rev Microbiol. 2018;16:745–759. doi: 10.1038/s41579-018-0089-x 30301974 PMC6278837

[3] Hajishengallis G , Chavakis T . Local and systemic mechanisms linking periodontal disease and inflammatory comorbidities. Nat Rev Immunol. 2021;21:426–440. doi: 10.1038/s41577-020-00488-6 33510490 PMC7841384

[4] Bläckberg A , Morenius C , Olaison L , et al. Infective endocarditis caused by HACEK group bacteria-a registry-based comparative study. Eur J Clin Microbiol Infect Dis. 2021;40:1919–1924. doi: 10.1007/s10096-021-04240-3 33852103 PMC8346386

[5] Fine DH , Markowitz K , Fairlie K , et al. A consortium of aggregatibacter actinomycetemcomitans, streptococcus parasanguinis, and filifactor alocis is present in sites prior to bone loss in a longitudinal study of localized aggressive periodontitis. J Clin Microbiol. 2013;51:2850–2861. doi: 10.1128/JCM.00729-13 23784124 PMC3754677

[6] Fine DH , Patil AG , Velusamy SK . Aggregatibacter actinomycetemcomitans (*Aa*) under the radar: myths and misunderstandings of aa and its role in aggressive periodontitis. Front Immunol. 2019;10:728. doi: 10.3389/fimmu.2019.00728 31040843 PMC6476972

[7] Belibasakis GN , Maula T , Bao K , et al. Virulence and pathogenicity properties of aggregatibacter actinomycetemcomitans. Pathogens. 2019;8:222. doi: 10.3390/pathogens8040222 31698835 PMC6963787

[8] Konig MF , Abusleme L , Reinholdt J , et al. Aggregatibacter actinomycetemcomitans-induced hypercitrullination links periodontal infection to autoimmunity in rheumatoid arthritis. Sci Transl Med. 2016;8:369–176. doi: 10.1126/scitranslmed.aaj1921 PMC5384717 27974664

[9] Baker JL , Mark Welch JL , Kauffman KM , et al. The oral microbiome: diversity, biogeography and human health. Nat Rev Microbiol. 2024;22:89–104. doi: 10.1038/s41579-023-00963-6 37700024 PMC11084736

[10] Nguyen CM , Kim JW , Quan VH , et al. Periodontal associations in cardiovascular diseases: the latest evidence and understanding. J Oral Biol Craniofac Res. 2015;5:203–206. doi: 10.1016/j.jobcr.2015.06.008 26587382 PMC4623887

[11] Dutzan N , Konkel JE , Greenwell-Wild T , et al. Characterization of the human immune cell network at the gingival barrier. Mucosal Immunol. 2016;9:1163–1172. doi: 10.1038/mi.2015.136 26732676 PMC4820049

[12] Hajishengallis G . New developments in neutrophil biology and periodontitis. Periodontol 2000. 2020;82:78–92. 31850633 10.1111/prd.12313

[13] Burn GL , Foti A , Marsman G , et al. The neutrophil. Immunity. 2021;54:1377–1391. doi: 10.1016/j.immuni.2021.06.006 34260886

[14] Brinkmann V . Neutrophil extracellular traps kill bacteria. Science. 2004;303:1532–1535. doi: 10.1126/science.1092385 15001782

[15] Wang H , Kim SJ , Lei Y , et al. Neutrophil extracellular traps in homeostasis and disease. Signal Transduct Target Ther. 2024;9:235. doi: 10.1038/s41392-024-01933-x 39300084 PMC11415080

[16] Doke M , Fukamachi H , Morisaki H , et al. Nucleases from prevotella intermedia can degrade neutrophil extracellular traps. Molecular oral microbiology. 2016;32(4). doi: 10.1111/omi.12171 PMC5516193 27476978

[17] Liu J , Sun L , Guo L , et al. A nuclease from streptococcus mutans facilitates biofilm dispersal and escape from killing by neutrophil extracellular traps. Front Cell Infect Microbiol. 2017;7:97. doi: 10.3389/fcimb.2017.00097 28401067 PMC5368189

[18] Baz AA , Hao H , Lan S , et al. Neutrophil extracellular traps in bacterial infections and evasion strategies. Front Immunol. 2024;15. doi: 10.3389/fimmu.2024.1357967 PMC10906519 38433838

[19] Boesze-Battaglia K , Alexander D , Dlakic M , et al. A journey of cytolethal distending toxins through cell membranes. Front Cell Infect Microbiol. 2016;6:81. doi: 10.3389/fcimb.2016.00081 27559534 PMC4978709

[20] Oscarsson J. , Claesson R. , Lindholm M. , et al. Tools of aggregatibacter actinomycetemcomitans to evade the host response. J Clin Med. 2019;8. 10.3390/jcm8071079 PMC6678183 31336649

[21] Elwell CA , Dreyfus LA . DNase I homologous residues in CdtB are critical for cytolethal distending toxin-mediated cell cycle arrest. Mol Microbiol. 2000;37:952–963. doi: 10.1046/j.1365-2958.2000.02070.x 10972814

[22] Shenker BJ , Hoffmaster RH , Zekavat A , et al. Induction of apoptosis in human T cells by actinobacillus actinomycetemcomitans cytolethal distending toxin is a consequence of G2 arrest of the cell cycle. J Immunol. 2001;167:435–441. doi: 10.4049/jimmunol.167.1.435 11418680

[23] Matangkasombut O , Wattanawaraporn R , Tsuruda K , et al. Cytolethal distending toxin from aggregatibacter actinomycetemcomitans induces DNA damage, S/G2 cell cycle arrest, and caspase- independent death in a saccharomyces cerevisiae model. Infect Immun. 2010;78:783–792. doi: 10.1128/IAI.00857-09 19995894 PMC2812194

[24] Fu Y , Maaβ S , du Teil Espina M , et al. Connections between exoproteome heterogeneity and virulence in the oral pathogen aggregatibacter actinomycetemcomitans. mSystems. 2022;7:e0025422. doi: 10.1128/msystems.00254-22 35695491 PMC9239275

[25] Hsu AY , Peng Z , Luo H , et al. Isolation of human neutrophils from whole blood and buffy coats. JoVE. 2021;e62837. doi: 10.3791/62837 34605812

[26] O’Toole GA . Microtiter dish biofilm formation assay. JoVE. 2011 e2437. doi: 10.3791/2437 PMC3182663 21307833

[27] Barrientos L , Marin-Esteban V , de Chaisemartin L , et al. An improved strategy to recover large fragments of functional human neutrophil extracellular traps. Front Immunol. 2013;4:166. doi: 10.3389/fimmu.2013.00166 23805143 PMC3690357

[28] Henneck T , Krüger C , Nerlich A , et al. Comparison of NET quantification methods based on immunofluorescence microscopy: hand-counting, semi-automated and automated evaluations. Heliyon. 2023;9:e16982. doi: 10.1016/j.heliyon.2023.e16982 37484269 PMC10361044

[29] Brinkmann V , Goosmann C , Kuhn LI , et al. Automatic quantification of *in vitro* NET formation. Front Immunol. 2012;3:413. 23316198 10.3389/fimmu.2012.00413 PMC3540390

[30] Huntley JF , Robertson GT , Norgard MV . Method for the isolation of francisella tularensis outer membranes. J Vis Exp. 2010 2044. doi: 10.3791/2044 20613713 PMC3156061

[31] Soh KY , Loh JMS , Proft T . Cell wall-anchored 5′-nucleotidases in gram-positive cocci. Mol Microbiol. 2020;113:691–698. doi: 10.1111/mmi.14442 31872460

[32] Garavaglia S , Bruzzone S , Cassani C , et al. The high-resolution crystal structure of periplasmic haemophilus influenzae NAD nucleotidase reveals a novel enzymatic function of human CD73 related to NAD metabolism. Biochem J. 2012;441:131–141. doi: 10.1042/BJ20111263 21933152

[33] Soh KY , Loh JMS , Proft T . Orthologues of streptococcus pyogenes nuclease A (SpnA) and streptococcal 5’-nucleotidase A (S5nA) found in streptococcus iniae. J Biochem. 2018;164:165–171. doi: 10.1093/jb/mvy039 29659850

[34] Qian M , Xu K , Zhang M , et al. 5’-Nucleotidase is dispensable for the growth of salmonella typhimurium but inhibits the bactericidal activity of macrophage extracellular traps. Arch Microbiol. 2022;205:20. doi: 10.1007/s00203-022-03353-3 36482126

[35] Bakari S , André F , Seigneurin-Berny D , et al. Lactococcus lactis: recent developments in functional expression of membrane proteins 2014. pp. 107–132.

[36] Davarpanah E , Seyed N , Bahrami F , et al. Lactococcus lactis expressing sand Fly PpSP15 salivary protein confers long-term protection against leishmania major in BALB/c mice. PLoS Negl Trop Dis. 2020;14:e0007939. doi: 10.1371/journal.pntd.0007939 31899767 PMC6941807

[37] Vitkov L , Singh J , Schauer C , et al. Breaking the gingival barrier in periodontitis. Int J Mol Sci. 2023;24:4544. doi: 10.3390/ijms24054544 36901974 PMC10003416

[38] Vitkov L , Klappacher M , Hannig M , et al. Extracellular neutrophil traps in periodontitis. J Periodontal Res. 2009;44:664–672. doi: 10.1111/j.1600-0765.2008.01175.x 19453857

[39] Urban CF , Ermert D , Schmid M , et al. Neutrophil extracellular traps contain calprotectin, a cytosolic protein complex involved in host defense against candida albicans. PLoS Pathog. 2009;5:e1000639. doi: 10.1371/journal.ppat.1000639 19876394 PMC2763347

[40] Buchanan JT , Simpson AJ , Aziz RK , et al. DNase expression allows the pathogen group A streptococcus to escape killing in neutrophil extracellular traps. Curr Biol. 2006;16:396–400. doi: 10.1016/j.cub.2005.12.039 16488874

[41] de Buhr N , Neumann A , Jerjomiceva N , et al. Streptococcus suis DNase SsnA contributes to degradation of neutrophil extracellular traps (NETs) and evasion of NET-mediated antimicrobial activity. Microbiology. 2014;160:385–395. doi: 10.1099/mic.0.072199-0 24222615

[42] Hirschfeld J , White PC , Milward MR , et al. Modulation of neutrophil extracellular trap and reactive oxygen species release by periodontal bacteria. Infect Immun. 2017;85. doi: 10.1128/IAI.00297-17 PMC5695129 28947649

[43] Hirschfeld J , White PC , Milward MR , et al. Effects of aggregatibacter actinomycetemcomitans leukotoxin on neutrophil migration and extracellular trap formation. J Oral Microbiol. 2016;8:33070. doi: 10.3402/jom.v8.33070 27834173 PMC5103672

[44] Bonilla MC , Lassnig S , Obando Corella A , et al. Studying the interaction of neutrophils and glaesserella parasuis indicates a serotype independent benefit from degradation of NETs. Pathogens. 2022;11:880. doi: 10.3390/pathogens11080880 36015001 PMC9415231

[45] de Buhr N , Bonilla MC , Pfeiffer J , et al. Degraded neutrophil extracellular traps promote the growth of actinobacillus pleuropneumoniae. Cell Death Dis. 2019;10:657. doi: 10.1038/s41419-019-1895-4 31506432 PMC6736959

[46] Fleischmann RD , Adams MD , White O , et al. Whole-genome random sequencing and assembly of haemophilus influenzae rd. Science. 1995;269:496–512. doi: 10.1126/science.7542800 7542800

[47] Kemmer G , Reilly TJ , Schmidt-Brauns J , et al. NadN and e (P4) are essential for utilization of NAD and nicotinamide mononucleotide but not nicotinamide riboside in haemophilus influenzae. J Bacteriol. 2001;183:3974–3981. doi: 10.1128/JB.183.13.3974-3981.2001 11395461 PMC95280

[48] Zagursky RJ , Burns DL , Ooi P , et al. Identification of a haemophilus influenzae-nucleotidase protein: cloning of the nucA gene and immunogenicity and characterization of the NucA protein. Infect Immun. 2000;68(5):2525–2534. doi: 10.1128/IAI.68.5.2525-2534.2000 10768940 PMC97455

[49] Zijnge V , Kieselbach T , Oscarsson J . Proteomics of protein secretion by aggregatibacter actinomycetemcomitans. PLoS One. 2012;7:e41662. doi: 10.1371/journal.pone.0041662 22848560 PMC3405016

[50] Schwechheimer C , Kuehn MJ . Outer-membrane vesicles from gram-negative bacteria: biogenesis and functions. Nat Rev Microbiol. 2015;13:605–619. doi: 10.1038/nrmicro3525 26373371 PMC5308417

[51] Jan AT . Outer membrane vesicles (OMVs) of gram-negative bacteria: a perspective update. Front Microbiol. 2017;8:1053. doi: 10.3389/fmicb.2017.01053 28649237 PMC5465292

[52] Kieselbach T , Zijnge V , Granström E , et al. Proteomics of aggregatibacter actinomycetemcomitans outer membrane vesicles. PLoS One. 2015;10:e0138591. doi: 10.1371/journal.pone.0138591 26381655 PMC4575117

[53] Fu Y , Trautwein-Schult A , Piersma S , et al. Characterization of outer membrane vesicles of aggregatibacter actinomycetemcomitans serotypes a, b and c and their interactions with human neutrophils. Int J Med Microbiol. 2025;319:151655. doi: 10.1016/j.ijmm.2025.151655 40424897

[54] Rompikuntal PK , Thay B , Khan MK , et al. Perinuclear localization of internalized outer membrane vesicles carrying active cytolethal distending toxin from aggregatibacter actinomycetemcomitans. Infect Immun. 2012;80:31–42. doi: 10.1128/IAI.06069-11 22025516 PMC3255663

[55] du Teil Espina M , Fu Y , van der Horst D , et al. Coating and corruption of human neutrophils by bacterial outer membrane vesicles. Microbiol Spectr. 2022;10:e00753-22. doi: 10.1128/spectrum.00753-22 36000865 PMC9602476

[56] Wang Z , Zhu D , Zhang Y , et al. Extracellular vesicles produced by avian pathogenic escherichia coli (APEC) activate macrophage proinflammatory response and neutrophil extracellular trap (NET) formation through TLR4 signaling. Microb Cell Fact. 2023;22:177. doi: 10.1186/s12934-023-02171-6 37689682 PMC10492386

[57] Ma F , Guo X , Fan H . Extracellular nucleases of streptococcus equi subsp. Zooepidemicus degrade neutrophil extracellular traps and impair macrophage activity of the host. ApEnM. 2016;83:e02468–16. 10.1128/AEM.02468-16 PMC5203633 27815272

[58] Chang A , Khemlani A , Kang H , et al. Functional analysis of streptococcus pyogenes nuclease A (SpnA), a novel group A streptococcal virulence factor. Mol Microbiol. 2011;79:1629–1642. doi: 10.1111/j.1365-2958.2011.07550.x 21231972

