# Supplementary material for: Aggregatibacter actinomycetemcomitans NadN contributes to neutrophil extracellular trap degradation
Source: J Oral Microbiol. 2026 Jul 6;18(1):2694756. doi: 10.1080/20002297.2026.2694756 (PMC13347837; doi:10.1080/20002297.2026.2694756)
Supplement: YP_Aa manuscript_Supplement_TF.pdf [file ZJOM_A_2694756_SM8270.pdf]

Supplementary figures

Aggregatibacter actinomycetemcomitans NadN contributes to Neutrophil Extracellular Trap degradation

Yaowapa Puanglai[a], Anongnard Kasorn[b], Oranart Matangkasombut[c,d], Dujduan Waraho-Zhmayev[e], Soraya Chaturongakul[f] and Fabien Loison[a]*

[a]*Department of Microbiology, Faculty of Science, Mahidol University, Bangkok, Thailand;* [b]*Department of Basic Medical Science, Faculty of Medicine Vajira Hospital, Navamindradhiraj University, Bangkok, Thailand;* [c]*Department of Microbiology and Center of Excellence on Oral Microbiology and Immunology, Faculty of Dentistry, Chulalongkorn University, Bangkok, Thailand;* [d]*Research Laboratory of Biotechnology, Chulabhorn Research Institute, Bangkok, Thailand;* [e]*Biological Engineering Program, Faculty of Engineering, King Mongkut's University of Technology Thonburi, Bangkok, Thailand ;* [f]*Molecular Medical Biosciences Cluster, Institute of Molecular Biosciences, Mahidol University, Nakhon Pathom, Thailand*

* Correspondence: Fabien Loison

Mail: fabien.loi@mahidol.ac.th Address: Department of Microbiology Faculty of Science, Mahidol University 272 Rama VI Road, Ratchathewi Bangkok 10400 THAILAND

Figure S1

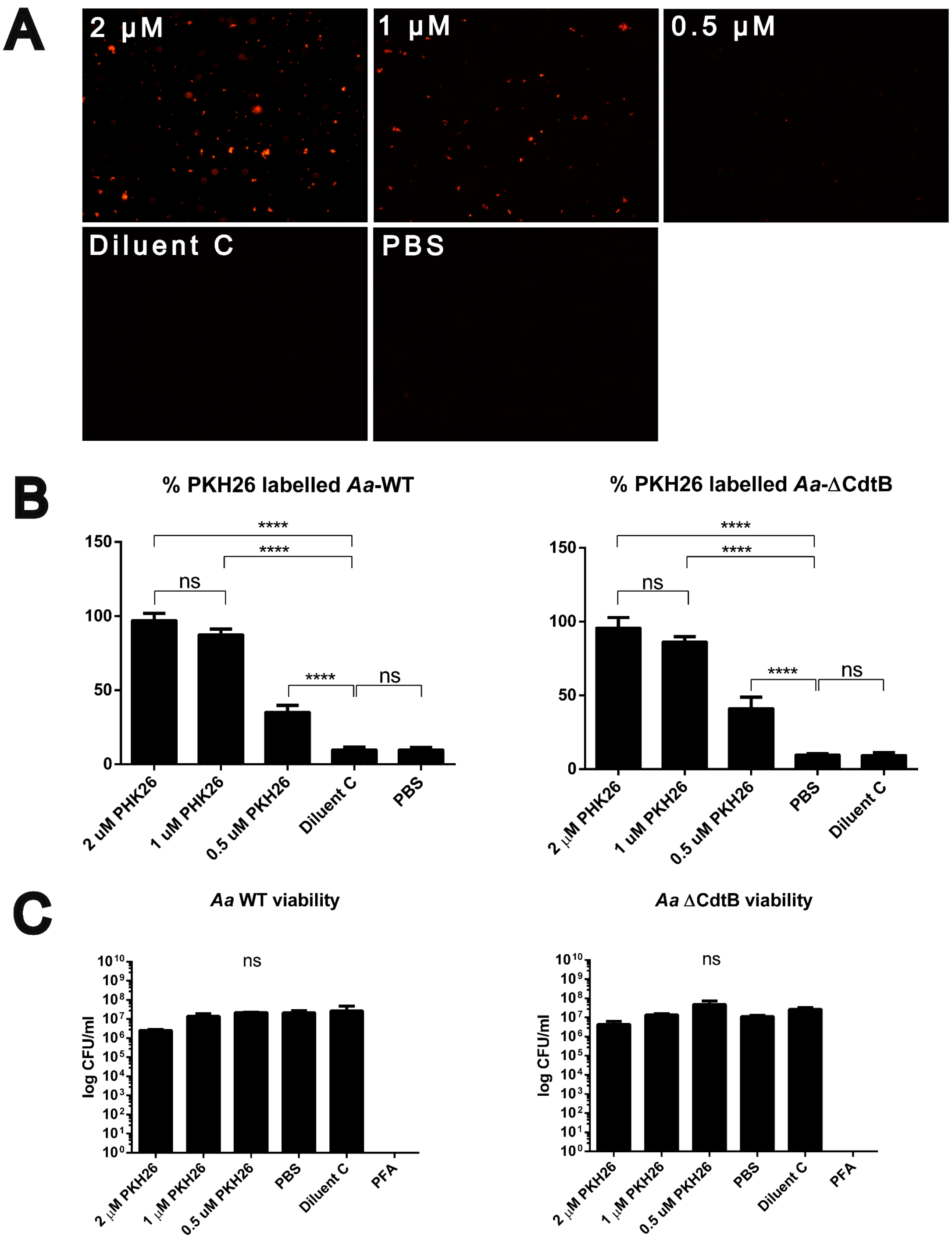

Figure S1 *A. actinomycetemcomitans* labeling with PKH26 fluorescent dye.

(A) Representative fluorescence microscopy images of *A. actinomycetemcomitans* following membrane labeling with varying concentrations of PKH26 (0.5, 1, and 2 μM). (Magnification: 400x). (B) Quantitative assessment of labeling efficiency, expressed as

the mean percentage of PKH26-positive bacteria, as detected by flow cytometry. (C) Evaluation of bacterial viability post-labeling. Survival was determined by quantifying colony-forming units (CFU) following PKH26 staining. Data were presented as mean +/- SD (n=3). Statistical significance was determined by one-way ANOVA followed by Tukey's multiple comparisons test; **** $p < 0.0001$; ns, non-significant.

Figure S2

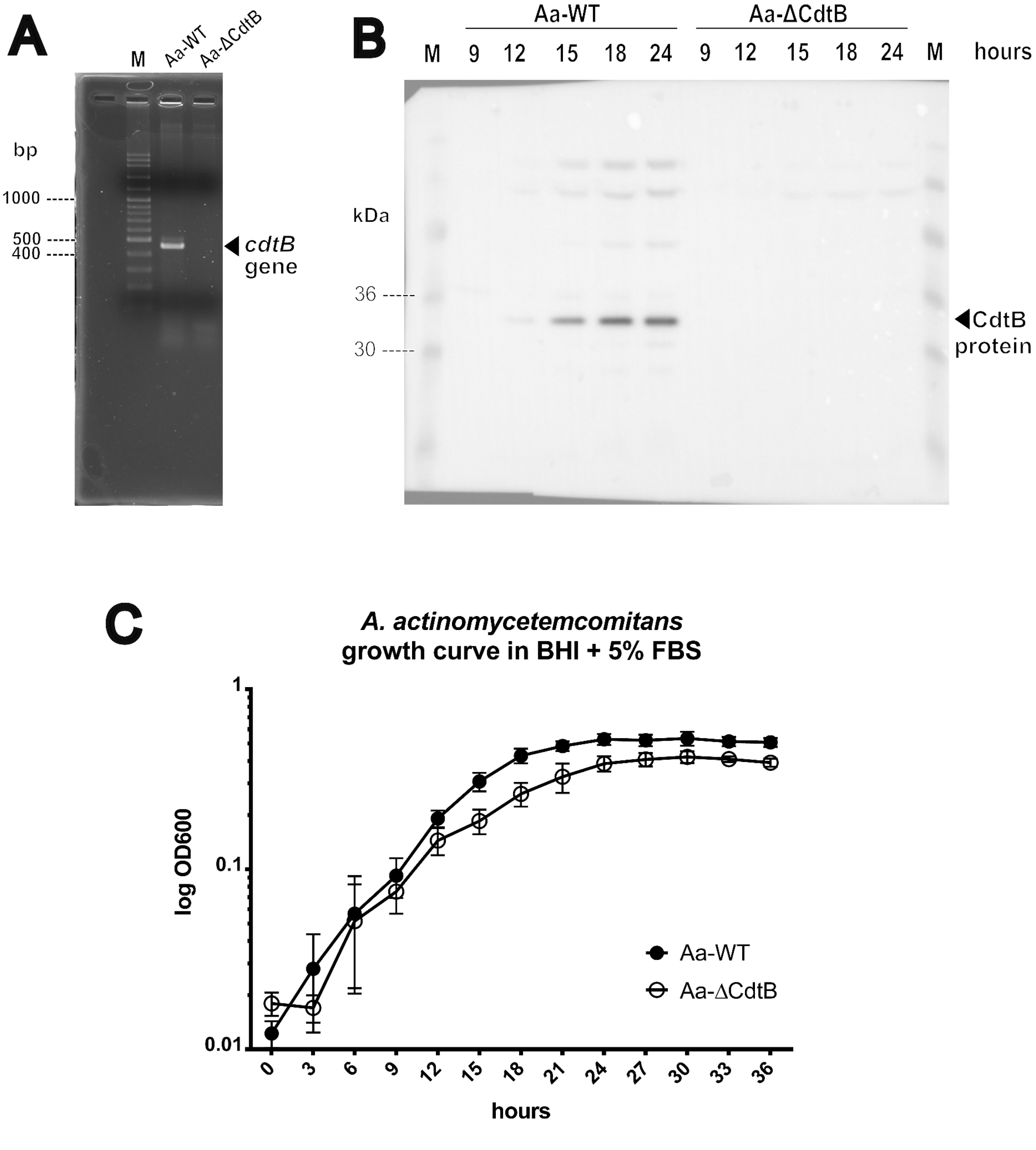

Figure S2 Characterization and growth kinetics of the *A. actinomycetemcomitans* WT and ΔCdtB mutant. (A) PCR amplification of cytolethal distending toxin B (*cdtB*) gene using genomic DNA templates. (B) Western blot analysis of CdtB (32 kDa) expression using CdtB-specific antibodies (rabbit anti-CdtB). M: Protein ladder. (C) Comparative growth kinetics of *A. actinomycetemcomitans* wild-type (WT) and ΔCdtB strains. Mean +/- SD, n=3.

Figure S3

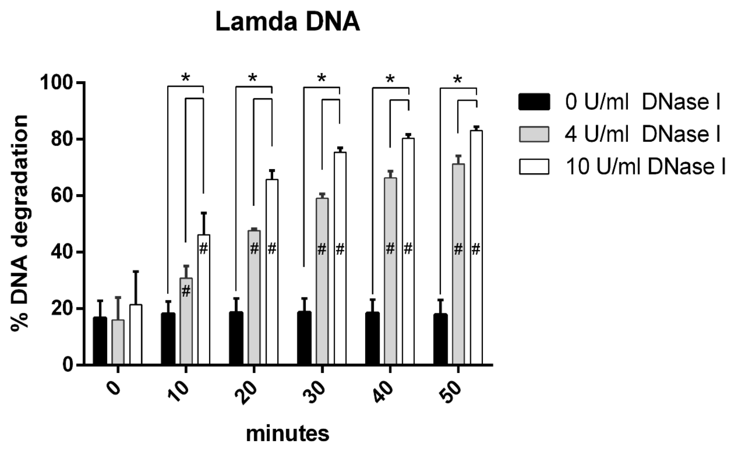

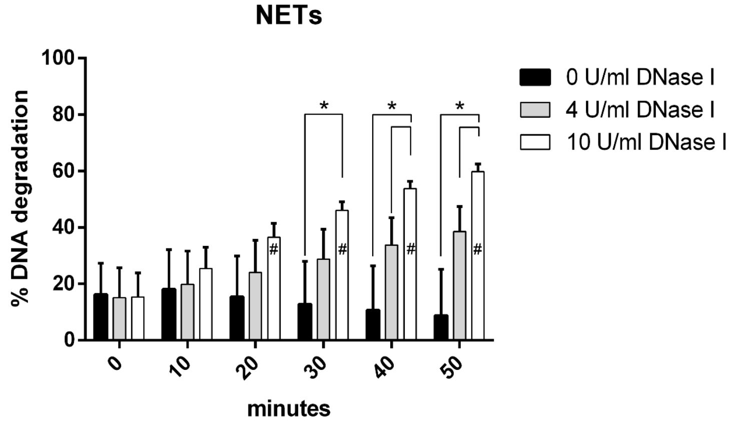

Figure S3. Quantification of DNase I-mediated degradation of lambda DNA and isolated NETs. Nuclease activity was assessed across varying concentrations of DNase I using a fluorescence-based microplate assay. Bars represent the mean +/- SD (n=3). Statistical significance was determined by multiple t-tests followed by the Holm-Sidak correction for multiple comparisons. (* $p < 0.05$ vs. the untreated control; # $p < 0.05$ vs. the corresponding DNase concentration at the t=0 baseline).

Figure S4

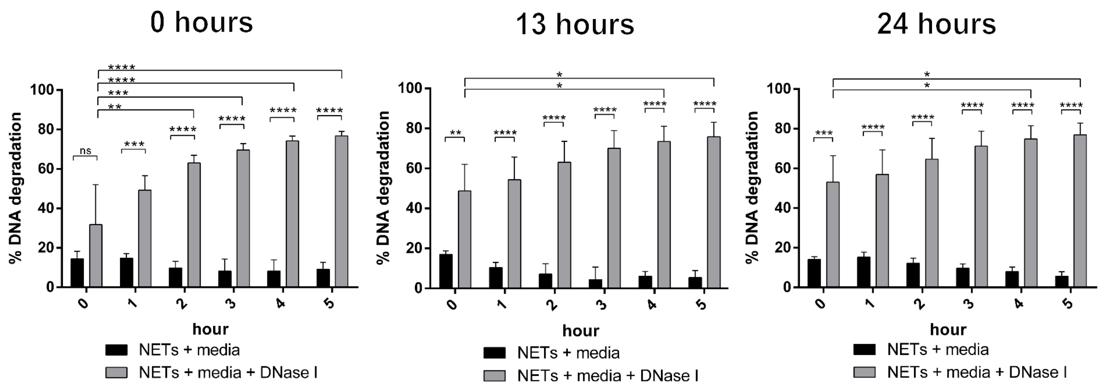

Figure S4. No DNase activity was identified in *A. actinomycetemcomitans* conditioned media. Cell-free RPMI supernatants were harvested from *A. actinomycetemcomitans*-WT cultures at 0, 13, and 24 hours, followed by concentration and incubation with isolated NETs for 6 hours at 37°C. NETs degradation was quantified using a PicoGreen fluorescence-based microplate assay. Black bars, conditioned media fractions; grey bars, conditioned media and DNase I (4 U/ml). Mean +/- SD, n=3. * $p < 0.05$, ** $p < 0.01$, *** $p < 0.0005$, **** $p < 0.0001$, two-way ANOVA followed by Tukey's post-hoc multiple comparisons test.

Figure S5

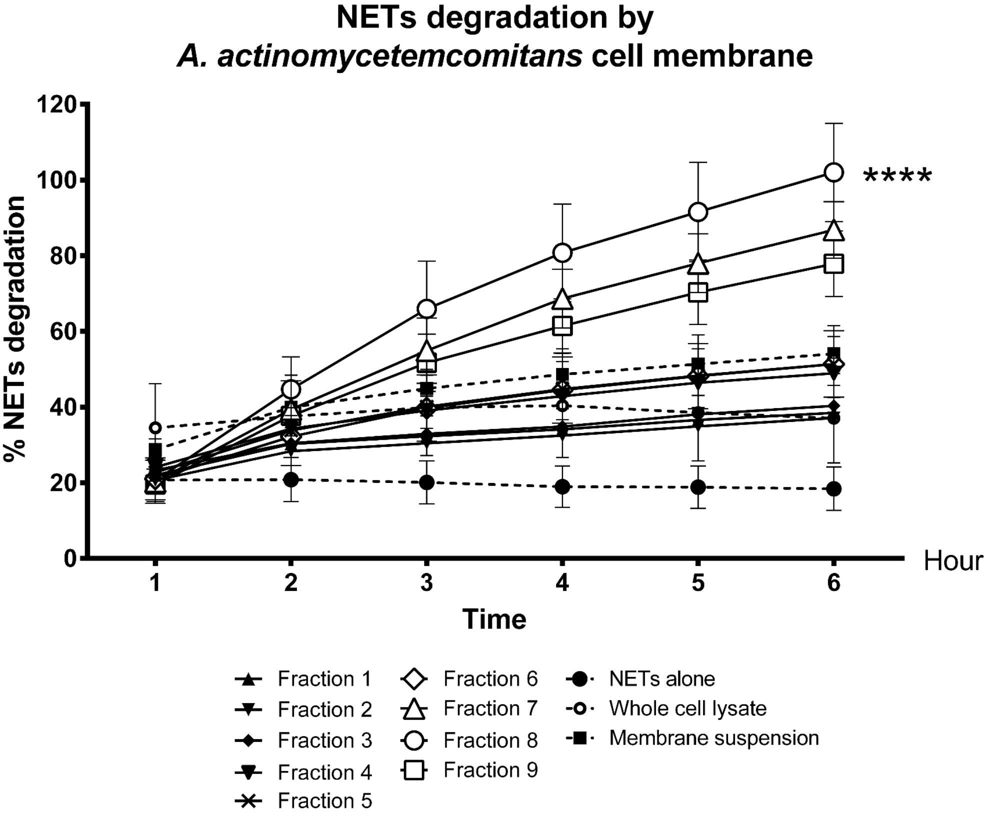

Figure S5. DNase activity was detected in *A. actinomycetemcomitans* membrane fractions. Isolated NETs were incubated with equal protein concentrations (150 ng) of *A. actinomycetemcomitans* whole-cell lysates, total membrane suspensions, or individual membrane fractions (Fractions 1–9) for 6 hours at 37°C. NETs degradation was quantified using a PicoGreen fluorescence-based microplate assay. Mean ± SEM (n=4). **** $p < 0.0001$, two-way ANOVA followed by Dunnett's multiple comparisons test.

Figure S6

Query_10001 WP_010922113.1 endonuclease/exonuclease/phosphatase family protein [Streptococcus pyogenes]
Query_10002 WP_005575874.1 NAD nucleotidase [Aggregatibacter actinomycetemcomitans]
Query_10003 WP_110426634.1 NAD nucleotidase [Haemophilus influenzae]
Query_10004 AAC21874.1 5'-nucleotidase, putative [Haemophilus influenzae Rd KW20]

```
Query_10001  1    MINKKCIIPVSLLTLAITLTSVEEVTSRQNLTYANEIVTQRPKRESVISDKSNFPVISPYLASVDFGERKTPLPTP-DKG  79
Query_10002  1    ---------------------------------------------MFTLKKTLTHILSTTFVFCSTSGLAANPPEDNHKA  35
Query_10003  1    ---------------------------------------------MLLSKKSATFALSVFAMLFTSVALAKEAPQA-HKA  34
Query_10004  1    ---------------------------------------------MLLSKKSASFALSAFAMLFTSVALAKEAPQA-HKA  34

Query_10001  80   VKVTTEQSIAQVRK-----GPEERPYTVTGKITSVINGWGGYGFYIQDSEGIGLYVYPQKDLGYSKGDIVQLTGTLTRFK  154
Query_10002  36   V----ELSILHINDHHSYLEPHEARILLNGKETKVNIG----GFSAVNSKLNELRKKYKNPLVLHAGDAITGTLYFTLFG  107
Query_10003  35   V----ELSILHINDHHSYLEPHETRINLNGQQTKVDIG----GFSAVNAKLNKLRKKYKNPLVLHAGDAITGTLYFTLFG  106
Query_10004  35   V----ELSILHINDHHSYLEPHETRINLNGQQTKVDIG----GFSAVNAKLNKLRKKYKNPLVLHAGDAITGTLYFTLFG  106

Query_10001  155  GDLQLQQVTAHKKLELSFPTSVKEAVISELETTTPSTLVKLSHVTVGELSTDQYNNTSFLVRDDSGKSIVVHIDHRTGVK  234
Query_10002  108  GSADAAVMNAG----------------------------NFHYFTLGNHEFDAGNEG--LLKLLEPLKIPVLSANVIPDK  157
Query_10003  107  GSADAAVMNAG----------------------------NFHYFTLGNHEFDAGNEG--LLKLLEPLKIPVLSANVIPDK  156
Query_10004  107  GSADAAVMNAG----------------------------NFHYFTLGNHEFDAGNEG--LLKLLEPLKIPVLSANVIPDK  156

Query_10001  235  GADVVTKISQGDLINLTAILSIVDGQLQLRPFSLEQLEVVKKVTSSNSDASSRNIVKIGEIQGASHTSPLLKKAVTVEQV  314
Query_10002  158  GSILYNKWKPYDIFS-------VNGE----KIGIIGLDTVNKTV--NSSSPGKDVKFYDEIATTQIMANALKAQGVNKII  224
Query_10003  157  SSILYNKWKPYDIFT-------VDGE----KIAIIGLDTVNKTV--NSSSPGKDVKFYDEIATAQIMANALKQQGINKII  223
Query_10004  157  NSILYNKWKPYDIFT-------VDGE----KIAIIGLDTVNKTV--NSSSPGKDVKFYDEIATAQIMANALKQQGINKII  223

Query_10001  315  VVTYLDDSTHFYVQDLNGDGDLATSDGIRVFAKNAKVQVGDVLTISGEVEEFFGRGYEERKQTDLTITQIVAKAVTKTGT  394
Query_10002  225  LLSHAGSGKNIEIAQKVNDID---------------------IIVTGDSHYLYGN--DELRELKLPVVYEY---------  272
Query_10003  224  LLSHAGSEKNIEIAQKVNDID---------------------VIVTGDSHYLYGN--DELRSLKLPVIYEY---------  271
Query_10004  224  LLSHAGSEKNIEIAQKVNDID---------------------VIVTGDSHYLYGN--DELRSLKLPVIYEY---------  271

Query_10001  395  AQVPSPLVLGKDRIAPANIIDNDGLRVFDPEEDAIDYWESMEGMLVAVDDAKILGPMKNKEIYVLPGSSTRPLNNSGGVL  474
Query_10002  273  ---------------PLEFKSPNGEPVFVMEAWA---YSAVVGDLGVKFSKDGIASIIRKTPHVLMSTNKLKVKNSDGNW  334
Query_10003  272  ---------------PLEFKNPNGEPVFVMEGWA---YSAVVGDLGVKFSPEGIASITRKIPHVLMSSHKLQVKNSEGKW  333
Query_10004  272  ---------------PLEFKNPNGDPVFVMEGWA---YSAVVGDLGVKFSPEGIASITRKIPHVLMSSHKLQVKNAEGKW  333

Query_10001  475  LPANSYNTDVIPVLFKKGKQIIKAGDSYKGRLAGPVSYSYGNYKVFVDDSKNMPSLMDGHLKPEKTNLQKDLSKLSIASY  554
Query_10002  335  QELS-------------GEERQKAIQSLQKMKS-----------VSLDYHDKKTDKLISKYRYEKDQLAKEVIGSIVGQ-  389
Query_10003  334  AELT-------------GDERKKALDTLKSMKS-----------ISLDDHDAKTDKLIAKYKSEKDRLAQEIVGVITGS-  388
Query_10004  334  TELT-------------GDERKKALDTLKSMKS-----------ISLDDHDAKTDMLISKYKSEKDRLAQEIVGVITGS-  388

Query_10001  555  NIENFSANPSSTKDEKVKRIAESFIHDLNAPDIIGLIEVQDNNGPTDDGTTDATQSAQRLIDAIKKLGGPTYRYVDIAPE  634
Query_10002  390  ------AMPGGSDNR-----------------------IPNKAGSNPEGSVATRFVAETMYNELKNVD------LVIQNA  434
Query_10003  389  ------AMPGGSANR-----------------------IPNKAGSNPEGSIATRFIAETMYNELKTVD------LTIQNA  433
Query_10004  389  ------AMPGGSANR-----------------------IPNKAGSNPEGSIATRFIAETMYNELKTVD------LTIQNA  433

Query_10001  635  NNVDGGQPGGNIRTGFLYQPERVSLSDKPKGGARDALTWVNGELNLSVGRIDPTNAAW---KDVRKSLAAEFIFQGRKVV  711
Query_10002  435  GGVRSDILPGDVTFNDAYTFLPFGNTLFTYKMEGSLIKQAL-EDALQFALVDGSTGGFPYGAGVRYEANETPNADGKRLV  513
Query_10003  434  GGVRADILPGNVTFNDAYTFLPFGNTLYTYKMEGSLVKQVL-EDAMQFALVDGSTGAFPYGAGIRYEANETPNAEGKRLV  512
Query_10004  434  GGVRADILPGNVTFNDAYTFLPFGNTLYTYKMEGSLVKQVL-EDAMQFALVDGSTGAFPYGAGIRYEANETPNAEGKRLV  512

Query_10001  712  VVANHLNSKRGDNALYGCVQPVTFKSEQRRHVLANMLAQFAKEGAKHQANIVMLGDFND--FEFTKTIQLIEEGDMVNLV  789
Query_10002  514  SVEV-FNKQTQ--------QWENIDDNKRYLVGTNSYIASGKDGYKTFGHL-----FNDPKYEGTDTY-LPDAESFIKFM  578
Query_10003  513  SVEV-LNKQTQ--------QWEPIDDNKRYLVGTNAYVAGGKDGYKTFGKL-----FNDPKYEGVDTY-LPDAESFIKFM  577
Query_10004  513  SVEV-LNKQTQ--------QWEPIDDNKRYLVGTNAYVAGGKDGYKTFGKL-----FNDPKYEGVDTY-LPDAESFIKFM  577

Query_10001  790  SRHDISDRYSYFHQGNNQTLDNILVSRHLLDHYEFDMVHVNSPFMEAHGRASDHDPLLLQLSFSKENDKAESSKQSVKAK  869
Query_10002  579  KKNPRFEAFKTSNVKLNVVSEALPKK------------------------------------------------------  604
Query_10003  578  KKHPHFEAYTSSNVKFNASTDALPKK------------------------------------------------------  603
Query_10004  578  KKHPHFEAYTSSNVKFNASTDALPKK------------------------------------------------------  603

Query_10001  870  KTSKGKLLPKTGDSLVYVITLLGTASLLVPILLLTKGKKES  910
Query_10002       -----------------------------------------
Query_10003       -----------------------------------------
Query_10004       -----------------------------------------
```

Figure S6. Multiple sequence alignment (MSA) of NadN homologs. Amino acid sequences of NadN from *A. actinomycetemcomitans* (Acc. No. WP_005575874.1; current study) and representative strains—*Streptococcus pyogenes* ATCC 700294 (Acc. No. WP_010922113.1), nontypeable *Haemophilus influenzae* strain P860259 (Acc. No. WP_110426634.1), and *H. influenzae* ATCC 51907D (Acc. No. AAC21874.1)—

were aligned using NCBI multiple protein sequence alignment. Red shade indicates the N-terminal region of the signal sequence. Hydrophobic regions are indicated in yellow colour. Blue colour shade denotes the C-terminal region of the signal sequence.

Figure S7

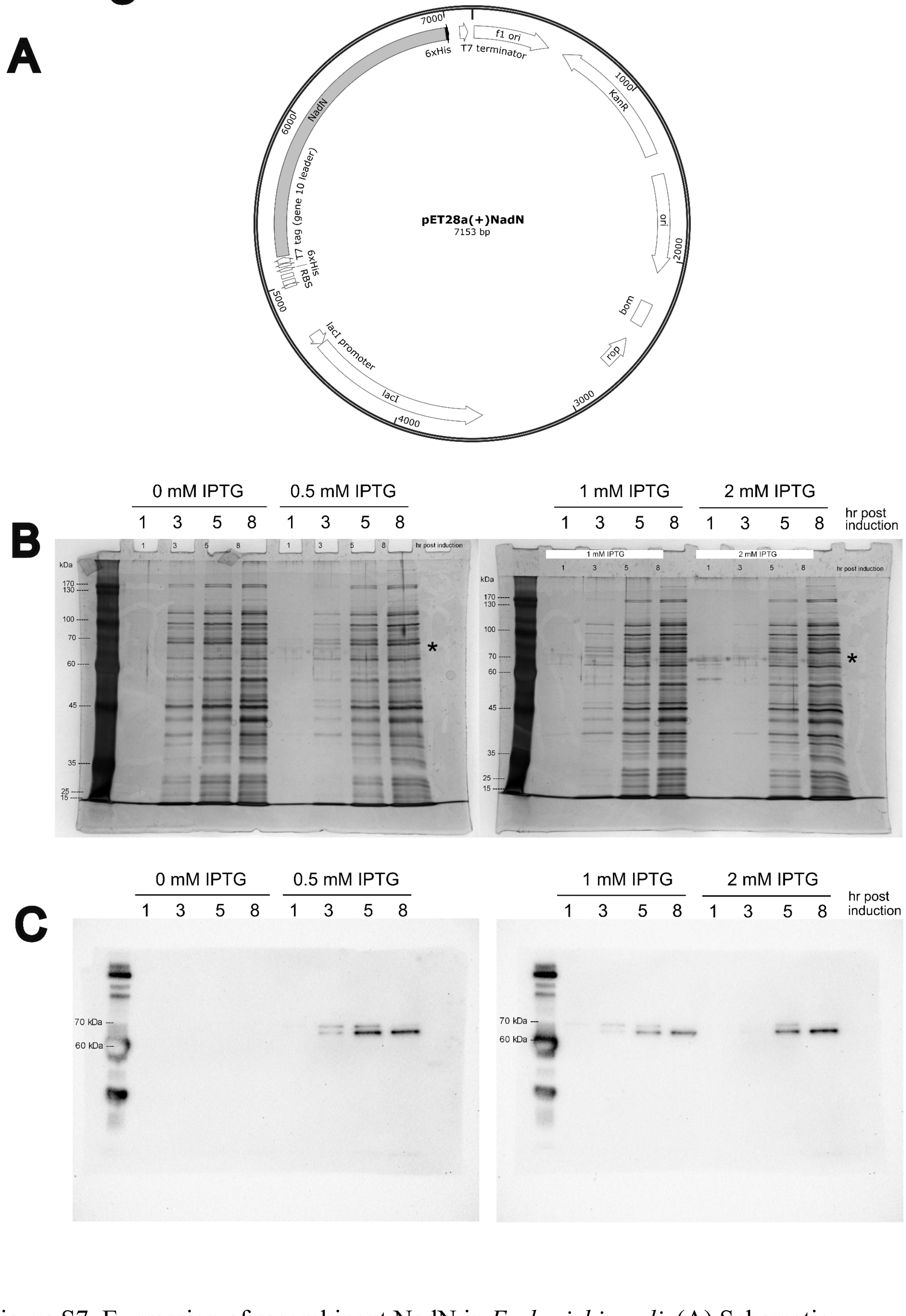

Figure S7. Expression of recombinant NadN in *Escherichia coli*. (A) Schematic representation of the pET28a-NadN-6xHis expression vector. (B) Optimization of rNadN expression in *E. coli* BL21(DE3). Cultures were induced with varying

concentrations of IPTG (0, 0.5, 1, and 2 mM), and whole-cell lysates were harvested at 1, 3, 5, and 8 hours post-induction. Proteins were separated by 10% SDS-PAGE and visualized via silver staining. Asterisks (*) indicated the migration bands corresponding to the predicted molecular weight of rNadN. (C) Western blot analysis of 6xHis-tagged rNadN within the soluble fraction of the crude lysate following IPTG induction, confirming successful protein expression and solubility.

Table S1 Protein concentration of conditioned RPMI medium

	Protein concentration (µg/ml)		
Culture time:	0 hour	13 hours	24 hours
Before concentration	1.2307	1.5334	1.5817
After concentration	11.4228	13.0573	13.7004

Table S2 Bacterial strains and plasmids used in this study

Bacterial strains or plasmids	Notable characteristics	Sources
A. actinomycetemcomitans strains		
Aa-WT	*Aa* wild type strain	Prof. Motoyuki Sugai
Aa-ΔCdtB	*Aa* deficient *cdtB* gene.	Prof. Motoyuki Sugai
E. coli strains		
E. coli DH5alpha	Plasmid cloning host and Gram-negative replicon	Purchased from NEB (#C2987H)
DH5alpha-pET28a-*NadN*	*E. coli* DH5alpha carrying pET28a-*NadN*, Km^r	This study
E. coli BL21(DE3)	*E. coli* plasmid-expressing host	Dr. Oranart Matangkasombut
E. coli BL21(DE3)-pET28a-*NadN*	*E. coli* BL21(DE3) carrying pET28a-*NadN*, Km^r	This study
Lactococcus lactis strains		
L. lactis NZ9000	Plasmid cloning host and Gram-positive replicon	Purchased from MoBiTec GmbH
L. lactis-pNZ8120	*L. lactis* NZ9000 carrying pNZ8120, Cm^r	This study
L. lactis-pNZ8120-*NadN*	*L. lacti*s NZ9000 carrying pNZ8120-*NadN*, Cm^r	This study
Plasmids		
pET28a(+)	*E. coli* gene-expressing vector, Km^r	Dr. Oranart Matangkasombut
pET28a-*NadN*	pET28a(+) containing *NadN* gene, Km^r	This study
pNZ8120	*L. lactis* gene-expressing vector, Cm^r	Purchased from MoBiTec GmbH
pNZ8120-*NadN*	pNZ8120 containing *NadN* gene, Cm^r	This study

Table S3 PCR primers used in this study

Primer	Sequence (5' to 3')	Restriction enzyme
Gst forward	ATGAAACTATATTTTAAACCG	-
Gst reverse	AATCAACCCTTCGGCTTTTTG	-
CdtB forward	GCTCCCGTCCAAATATGG	-
CdtB reverse	TGCTCTCGACGTGGTAAATG	-
NadN forward	ATCTATGCC/GGCGAATTCATGTTCACCCTG AAGAAAAC	Nae I
NadN reverse	TATATAT/CTAGATCAGTGGTGGTGGTGGTG GTGCTCGAG	Xba I
